# Synthesis of titanium nitride via hybrid nanocomposites based on mesoporous TiO_2_/acrylonitrile

**DOI:** 10.1038/s41598-021-84484-3

**Published:** 2021-03-03

**Authors:** Claudiu Locovei, Anita-Laura Chiriac, Andreea Miron, Sorina Iftimie, Vlad-Andrei Antohe, Andrei Sârbu, Anca Dumitru

**Affiliations:** 1 Faculty of Physics, University of Bucharest, 077125 Măgurele, Romania; 2 National Institute of Materials Physics (NIMP), 077125 Măgurele, Romania; 3 National Research and Development Institute for Chemistry and Petrochemistry (INCDCP-ICECHIM), Advanced Polymer Materials and Polymer Recycling, 060021 Bucharest, Romania; 4 Institute of Condensed Matter and Nanosciences (IMCN), Université catholique de Louvain (UCLouvain), 1348 Louvain-la-Neuve, Belgium

**Keywords:** Ceramics, Composites, Organic-inorganic nanostructures

## Abstract

In the present study, the synthesis of titanium nitride (TiN) by carbothermal reduction nitridation (CRN) reaction using nanocomposites made of mesoporous TiO_2_/acrylonitrile with different content of inorganic phase were explored. The choice of hybrid nanocomposite as precursor for the synthesis of TiN was made due to the possibility of having an intimate interface between the organic and inorganic phases in the mixture that can favours CRN reaction. Subsequently, the hybrid composites have been subjected to four-step thermal treatments at 290 °C, 550 °C, 1000 °C and 1400 °C under nitrogen atmosphere. The XRD results after thermal treatment at 1000 °C under nitrogen flow show the coexistence of two crystalline phases of TiO_2_, i.e. anatase and rutile, as well as TiN phase, together with the detection of amorphous carbon that proved the initiation of CRN reaction. Furthermore, the observations based on XRD patterns of samples thermally treated at 1400 °C in nitrogen atmosphere were in agreement with SEM analysis, that shows the formation of TiN by CRN reaction via hybrid nanocomposites mesoporous TiO_2_/acrylonitrile.

## Introduction

Titanium nitrides (TiN) have been researched extensively due their wide range of interesting properties such as high hardness, high thermal conductivity, high temperature stability and good corrosion resistance^1–3^. In the recent years, different strategies were used for the synthesis of TiN nanomaterials, such as: (1) nitridation of Ti-metal or titanium dioxide (TiO_2_) with various nitrogen sources (nitrogen or ammonia)^2^^,^^4^^,^^5^, (2) reaction of titanium isopropoxide with anhydrous hydrazine^6^, (3) reactive ball milling of titanium powders and urea at room temperature^7^, (4) two-step transition metal halide approach at relatively low temperatures of 400 °C^3^, (5) self-propagating high-temperature synthesis of cold-pressed titanium powder^8^ as well as (6) carbothermal reduction of titanium oxide under nitrogen flow^9–11^. One of the mostly reported strategies for the synthesis of TiN nanostructures is the direct nitridation of Ti/TiO_2_/TiCl_4_ precursor in ammonia atmosphere, which is an environmentally unfriendly method due to the use of dangerous and highly corrosive ammonia as nitrogen source^2^^,^^4^^,^^3^. As a consequence, different ammonia-free methods were developed for the synthesis of TiN such as hydrazide sol–gel method with subsequent high temperature calcination process^6^, synthesis of TiN@N-doped carbon composites from TiCl_4_ dissolved in ionic liquids like 1-butyl-3-methyl-pyridinium dicyanamide as nitrogen/carbon source^12^, reaction of metallic sodium with ammonium fluotitanate in an autoclave at 650 °C^13^, as well as reaction of titanium oxide with sodium amide at 500–600 °C for 12 h in an autoclave^14^. However, despite the relatively low temperatures range used for TiN synthesis, these methods still have some limitations, mostly related either to the expensive ionic liquids required, or to the extremely poisonous and dangerous reactants used as nitrogen source^1^. In this regard, the development of economical and environmentally friendly preparation route of TiN is highly desirable. As an ammonia free method, carbothermal reduction nitridation (CRN) reaction of titanium oxide in the presence of carbon and under nitrogen flow has some advantages such as the inexpensive nature of raw materials and the versatility of the carbon precursors^9–11^. Previous researchers have been used carbon black^10^^,^^15^ and activated carbon^9^^,^^11^ as carbon source for CRN process. As reported, in the synthesis of TiN by carbothermal reduction nitridation of TiO_2_, the excess of carbon and a good dispersion of TiO_2_ in the starting mixture, along with more intimate contact of the reactants, positively influence the reaction rate and significantly accelerate the reaction^15^. On the other hand, polymer materials that are converted into ceramic materials through controlled pyrolysis have been successfully investigated due to the possibility to adjust the phase composition, microstructure and morphology of the ceramic materials^16–19^. Based on the above considerations, in the present study, nanocomposites based on mesoporous TiO_2_/acrylonitrile were explored for the synthesis of TiN by CRN reaction. In our case, the polymer material was chosen due to the ability of polyacrylonitrile (PAN) to convert into carbon materials, when it is thermally treated under controlled atmosphere. PAN was exploited to obtain a carbon layer on pre-synthesized mesoporous TiO_2_^20^. The use of hybrid nanocomposites based on mesoporous TiO_2_/acrylonitrile could favour CRN reaction due to the high specific surface area that lead to a better interface between the organic and inorganic phases. Hybrid nanocomposite materials were obtained by radical polymerization of a vinyl monomer in pre-synthesized mesoporous TiO_2_ and further used as starting materials for the synthesis of TiN by CRN reaction at 1400 °C under nitrogen gas flow.

## Results and discussions

### Fourier Transform Infrared spectroscopy (FTIR) analysis

**Figure 1:**
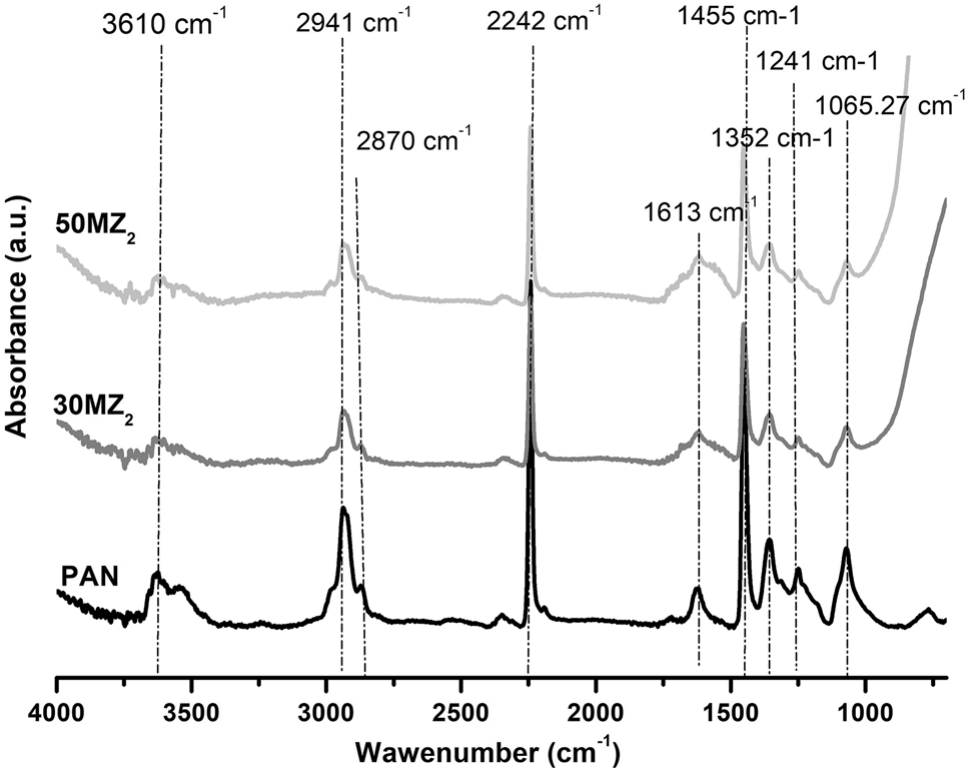
FTIR spectra of PAN, 30MZ_2_ and 50MZ_2_.

Figure 1 show FTIR spectra of synthesized PAN, 30MZ_2_ and 50MZ_2_. As seen in Fig. 1, the main characteristic bands of PAN spectra were assigned as follows: the band around 3610 cm^−1^ corresponds to O–H stretching, bands at 2941 cm^−1^ and 2870 cm^−1^ are assigned to symmetric and asymmetric of C–H bond stretching in polymer, respectively, absorption in the range between 2150 cm^−1^ and 2290 cm^−1^ are assigned to nitrile (–C$\equiv$ N) stretching vibration of the acrylonitrile unit in the polymer chains, the wide absorbance band around 1580–1620 cm^−1^ is attributed to –C=C– and –C=N– groups, 1455 cm^−1^ is a characteristic of the bending vibration of C-H in the CH_2_ group, 1352 cm^−1^ is a characteristic of the aliphatic CH groups along the PAN backbone, 1241 cm^−1^ and 1065.27 cm^−1^ are attributed to the skeleton vibration of the C–C and C–N groups, respectively^21–24^. In the case of hybrid nanocomposites, all the specific features of PAN are visible. Comparing with PAN, in the FTIR spectra of hybrid nanocomposites a decrease in the intensity of the characteristic absorption bands can be observed, due to the penetration of the polymer into the pores of the mesoporous inorganic host^23^.

### Thermal stability study

**Figure 2:**
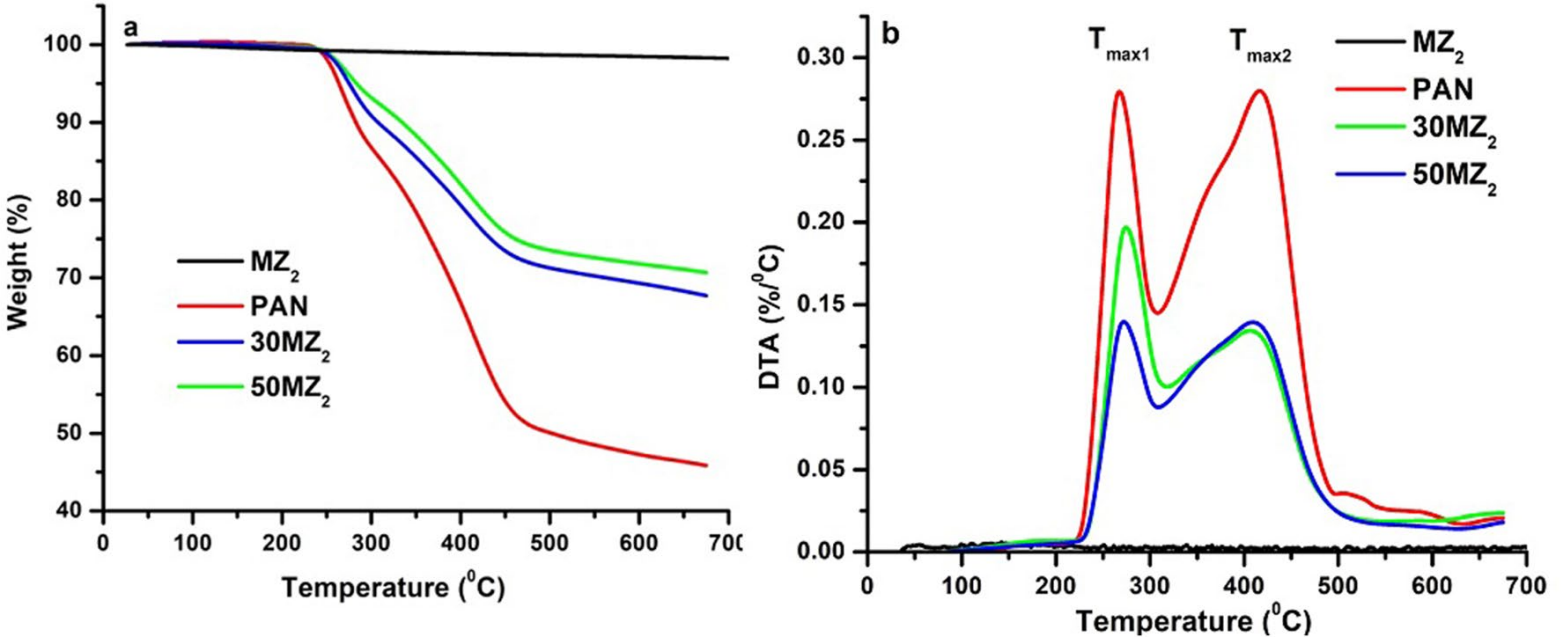
(**a**) TGA curves and (**b**) DTA curves of MZ_2_, PAN, 30MZ_2_ and 50MZ_2_.

TGA measurements, performed under nitrogen atmosphere, highlighted the influence of the mesoporous inorganic host upon the polymer’s thermostability which is highly important for upper stated applications. Figure 2a presents the TGA plots of hybrid nanocomposites, 30MZ_2_ and 50MZ_2_, together with TGA curves of PAN and mesoporous TiO_2_ (indexed as MZ_2_), in the studied range of temperature. Based on the TGA results, the stages of thermal treatment chosen in the present work for the conversion of hybrid nanocomposites to TiN were established.

**Figure 3:**
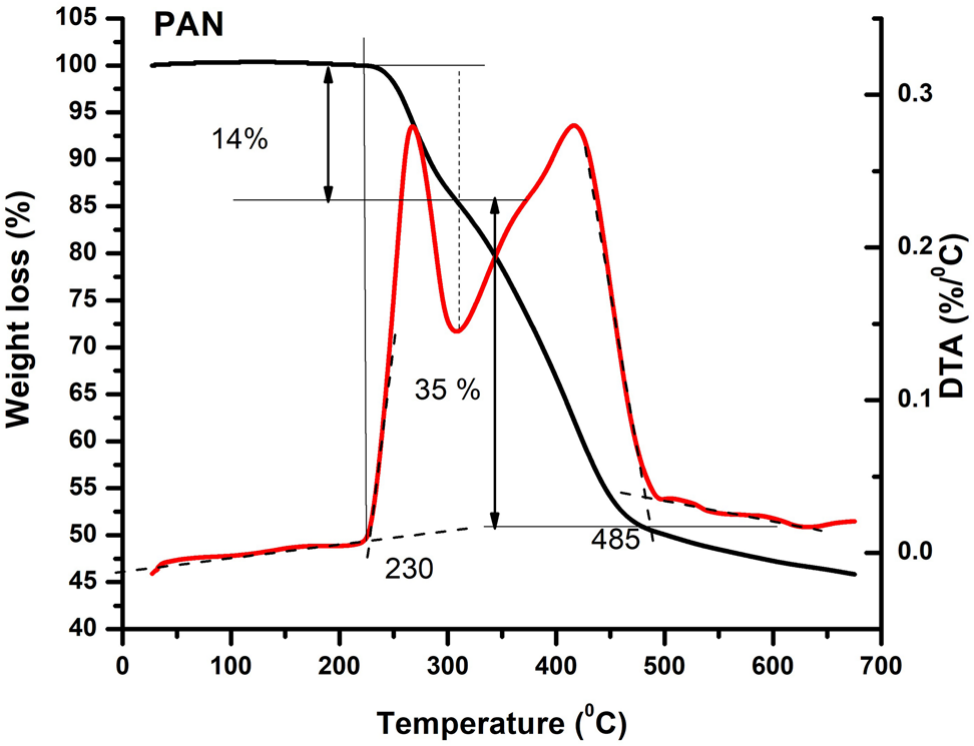
TGA and DTA curves of PAN.

TGA curves of mesoporous TiO_2_ (see Fig. 2a) show only a slight weight loss (2.44%) associated with a drying process-free bounded water loss, indicating the stability of TiO_2_^23^,^25^. All other samples presented four degradation stages, characteristic to the thermal degradation of PAN^26–29^. The first step in the TGA curve of PAN (see Figs. 2, 3) is located between 50 °C and 230 °C with a weight change lower than 2%, which should correspond to the loss of physically absorbed water and/or remaining monomer being present in the polymer. PAN sample remains relatively stable with a slight weight loss till 230 °C, followed by a faster degradation between 230 °C and 485 °C, which corresponds to the cyclization and pre-carbonization processes^26^,^28^. Total weight loss at 700 °C for this sample reached 54.1%. The most important mass loss ~39% takes place between 230 °C and 485 °C and involves two stages, as can be seen in the DTA curve from Fig. 2b. For the first stage located between 230 °C and 310 °C, with the maximum temperature T_max1_ of 267 °C, the weight loss is associated with cyclization of nitrile groups of PAN molecular chain, representing 14% of the weight loss and it can proceed in either an inert or oxidizing atmosphere^26^,^27^^,^^29^. The second stage with a maximum temperature T_max2_ of 417.16 °C and a weight loss of 35% is correlated with the formation of an aromatic structure and partial evaporation of HCN and vinyl acetonitrile^29^. Finally, within the fourth stage, in the temperatures range of 550 °C and 700 °C, a monotonous decrease of weight loss is observed, corresponding to a mass change of ~6%, which may come from the elimination of polymer chain fragments^26^.

The obtained hybrid nanocomposite materials have similar patterns with PAN thermogram showing all the four steps of thermal degradation presented above. Compared to the polyacrylonitrile used as reference, with a weight loss of 54.14%, the composites synthesized from PAN and MZ_2_ show lower decomposition values, due in part to the thermal stability of the mesoporous inorganic matrix. However, weight loss of polymeric hybrids is far from the theoretical organic content (50% or 70% AN). This significant difference in mass loss suggests that the polymer was constricted in the cavities of titanium oxide, supporting the hypothesis that PAN was incorporated into its pores. Table 1 shows the temperatures for the maxima of the main two stages of decomposition for PAN and hybrid nanocomposites appearing in Fig. 2b.

**Table 1:** Data obtained from TGA and DTA curve of MZ_2_, PAN, 30MZ_2_ and 50MZ_2_.

Sample	T_max1_ (°C)	T_max2_ (°C)	Weight loss (%)
MZ_2_	–	–	2.4
PAN	268	417	54.1
30MZ_2_	275	409	32.4
50MZ_2_	273	410	29.4

### Morpho-structural characterization

**Figure 4:**
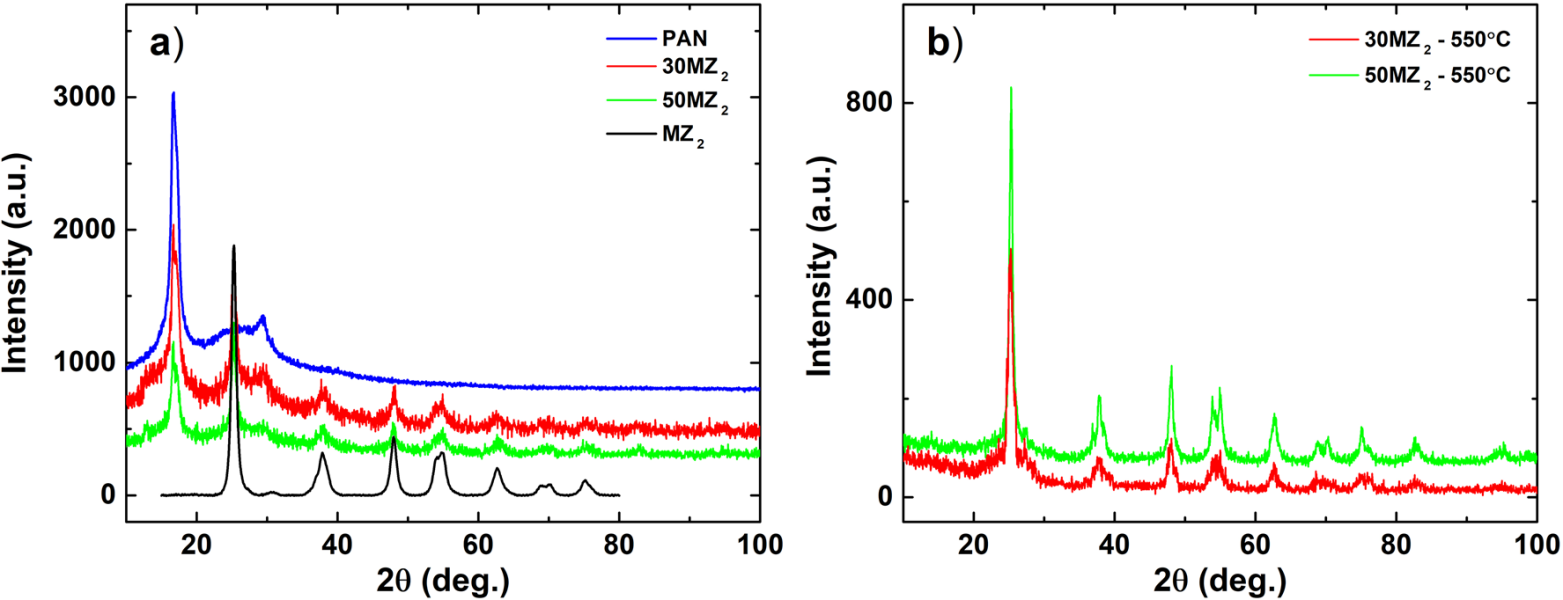
XRD patterns of (**a**) initial PAN, MZ_2_, 30MZ_2_ and 50MZ_2_; (**b**) hybrid nanocomposites thermally treated at 550 °C.

Figure 4 shows the XRD characterizations of initial samples MZ_2_, PAN, 30MZ_2_ and 50MZ_2_ (Fig. 4a), hybrid nanocomposites thermally treated at 550 °C (Fig. 4b), respectively. As shown in Fig. 4a, the XRD pattern of PAN presents two phases, one attributed to the crystalline phase of PAN and the other one represented by a broad scattering with maximum around 2$\theta$ = 25.5° is assigned to the amorphous molecular chain as reported elsewhere^26^^,^^30^^,^^31^. The XRD data (acquired in 2$\theta$ range of 15°–80°) of the mesoporous TiO_2_ show peaks that correspond only to the crystallographic planes of TiO_2_ anatase phase (ICDD 00-021−1272). XRD analysis of initial samples (Fig. 4a), 30MZ_2_ and 50MZ_2_, revealed the structural characteristics of both PAN and TiO_2_, highlighting the formation of the hybrid nanocomposite. After the thermal treatment at 550 °C, XRD patterns of hybrid nanocomposite (Fig. 4b) show that peaks attributed to PAN vanished and a broad peak corresponding to amorphous graphite (G) can be seen around 2$\theta$ = 26°, while the diffraction peaks of TiO_2_ anatase phase are more defined compared to the initial samples. These observations are in agreement with literature data^20^^,^^23^ and highlight the pre-carbonization process sustained by the thermal treatment, also seen in TGA results.

**Figure 5:**
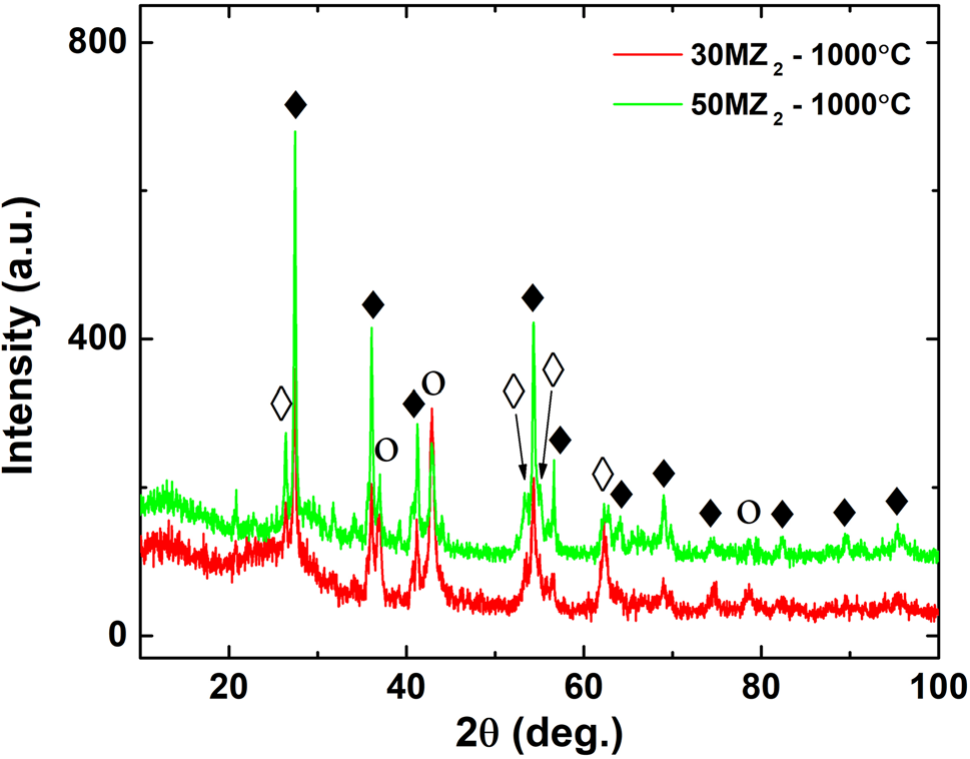
XRD patterns of 30MZ_2_ and 50MZ_2_ thermal treated at 1000 °C in nitrogen atmosphere; where $\blacklozenge$- TiO_2_ rutile, $\lozenge$-TiO_2_ anatase, $\circ$- TiN crystalline phases.

Figure 5 presents the diffraction patterns of 30MZ_2_ and 50MZ_2_ after the thermal treatment at 1000 °C under nitrogen atmosphere. XRD patterns of both samples show the presence of TiO_2_ rutile (ICDD 00-021−1276) and TiN (ICDD 00-038−1420) phases along with a broad peak attributed to the amorphous with the amorphous graphite phase with an additionally broad peak located approximately at 2$\theta$ = 14 °. Taken in consideration the initial composition of the hybrid nanocomposites together with the reactions involved during thermally treatments, the broad signal is attributed to trace of amorphous graphite oxide (GO)^32^. According to these results, this stage of the thermal treatment leads to the conversion of TiO_2_ anatase phase into TiO_2_ rutile phase and to the initiation of CRN reaction that is highlighted by the presence of TiN phase in the diffraction pattern. The intensity ratio between the corresponding diffraction planes of the two samples revealed that the CRN process is more advanced in the sample with lower content of inorganic phase. This fact may come from the lower concentration of the TiO_2_ anatase in the hybrid nanocomposite which leads to its more uniform and isotropic distribution, thus providing a superior dispersion of PAN in the inorganic matrix, as discussed later.

**Figure 6:**
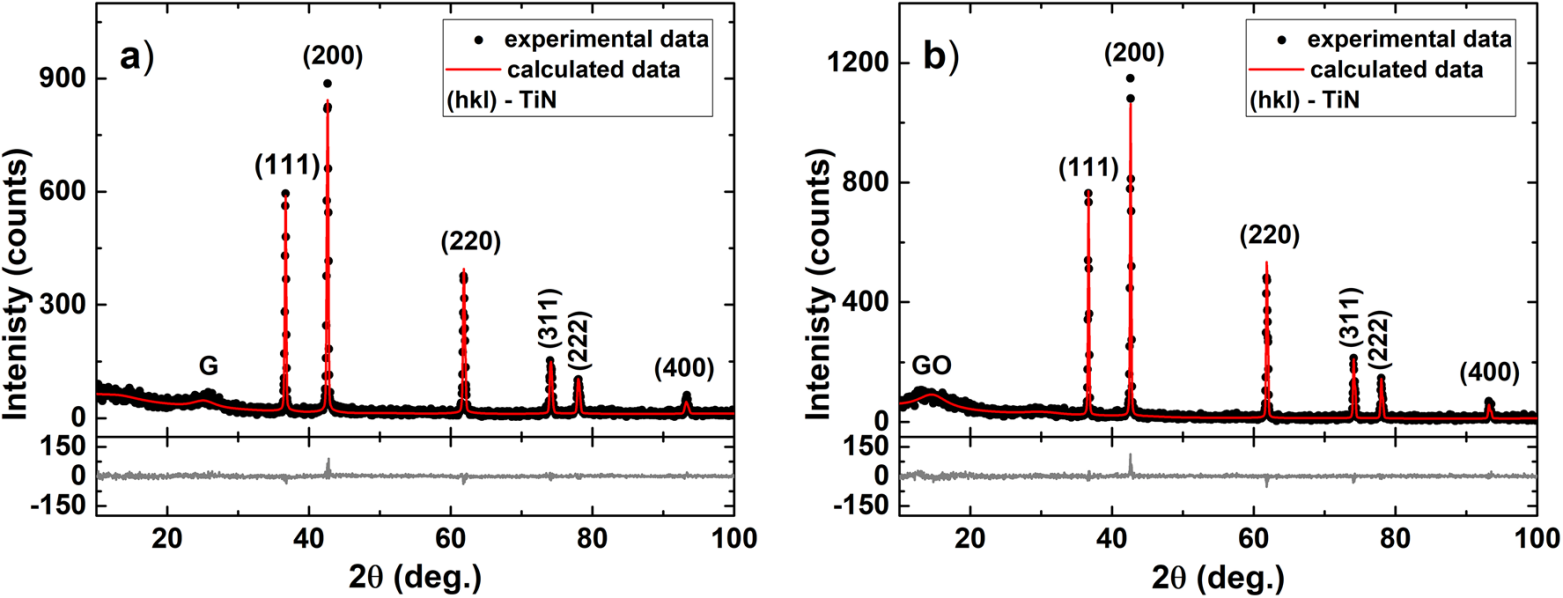
XRD experimental data with Rietveld refinement of (**a)** 30MZ_2_ and (**b**) 50MZ_2_ after thermal treatment at 1400 °C under nitrogen flow.

The plots of the data acquired in XRD characterization onto both samples after last stage of thermal treatment are presented in Fig. 6, together with Rietveld analysis^33^, respectively residuals, that were performed on the experimental data to refine the structural parameters. XRD patterns of 30MZ_2_ (Fig. 6a) and 50MZ_2_ (Fig. 6b), show mainly the crystalline structure of the TiN. However, for both samples some traces of amorphous graphite and amorphous graphite oxide are also observed as two broad peaks with different intensity ratio, apart from the diffraction planes of TiN phase. Amorphous graphite phase is more pronounced in 30MZ_2_ sample due to the organic/inorganic ratio in hybrid nanocomposite, leading to a higher content of carbon which was not entirely reduced to carbon monoxide and carbon dioxide in the high-temperatures regime^34^. Instead, in 50MZ_2_ sample, the ratio between TiO_2_ and PAN favored a better reduction rate of the carbon. However, in this case, the dispersion of PAN in the inorganic matrix is not as good as in 30MZ_2_, as it was observed in the XRD patterns of samples treated at 1000 °C. Thus, the presence of amorphous graphite oxide phase may come from an oxidation reaction of graphite at low temperatures during cooling process of the samples. Because cooling routine after thermal treatments was made passively, temperature drop rate was substantially greater at higher temperatures than cooling rates for lower temperatures. Taking into account the above considerations, for Rietveld analysis of 30MZ_2_ sample, an amorphous graphite phase with trace of amorphous graphite oxide was considered, besides TiN crystalline and amorphous phases. The ratio of G/GO was inverted for the 50MZ_2_ sample as compared to 30MZ_2_ sample. Graphite content that remained after the high-temperature thermal treatments was converted to amorphous graphite oxide at low temperatures in a higher amount for the 50MZ_2_ sample due to the greater oxygen concentration that came from the TiO_2_/PAN ratio, together with a poor dispersion of the organic material in the inorganic matrix. In order to increase the purity of TiN final product, some parameters such as the dispersion of hybrid nanocomposites, thermal treatments time and inorganic/organic ratio can be improved.

The structural parameters of TiN obtained from Rietveld refinement performed in MAUD program are shown in Table 2. The quantitative characterization employed the Rietveld lattice constant (*a*) and microstructure analysis. The higher value of crystallite size ($D_{ef}$) and smaller microstrain ($<\varepsilon ^{2}>^{1/2}$) in the 50MZ_2_ sample highlighted the distribution of mesoporous TiO_2_ and PAN in the hybrid nanocomposite. Thus, a soldering process between TiO_2_ particles occurs in thermal treatment at 1400 °C, which reduces the specific surface area of interaction with carbon and favouring the appearance of larger crystallites. Due to the small specific surface area of 50MZ_2_ hybrid nanocomposite in the desorption process of remaining oxygen at lower temperature, fewer structural defects have been created during the formation of amorphous graphite oxide. In comparison, 30MZ_2_ sample, with a finer dispersion presents smaller crystallite size and a bigger value of microstrain due to the higher specific surface area of interaction in hybrid the nanocomposite.

**Table 2:** Structural parameters taken from Rietveld refinement of TiN for samples thermal treated at 1400 °C.

Sample	$D_{ef}$ (nm)	$<\varepsilon ^{2}>^{1/2}$	*a* (Å)
30MZ_2_	154±9	1.2$\cdot$10^-3^±0.5$\cdot$10^-4^	4.24±1$\cdot$10^-4^
50MZ_2_	196±9	7.2$\cdot$10^-4^±0.3$\cdot$10^-4^	4.24±0.7$\cdot$10^-4^

**Figure 7:**
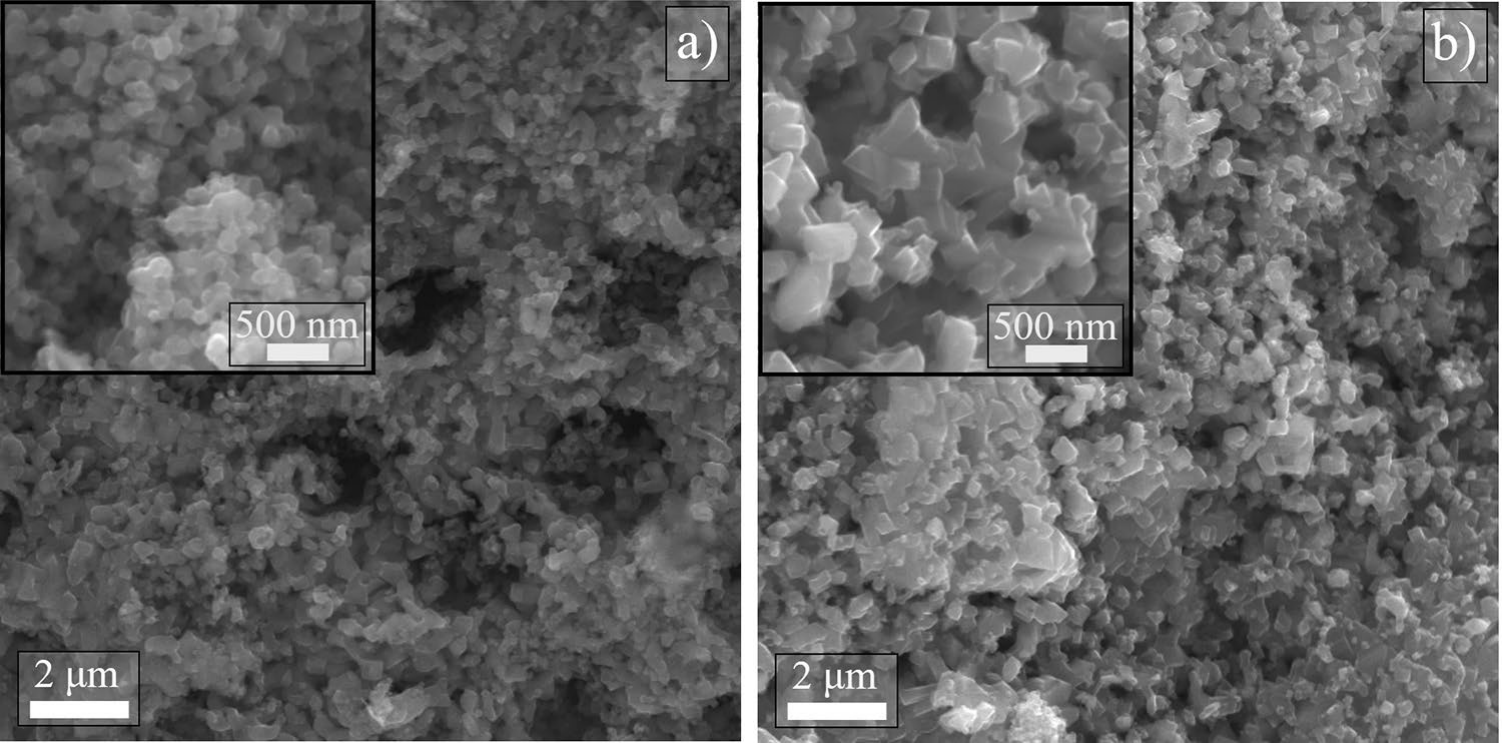
SEM micrographs of the sample (**a**) 30MZ_2_ and (**b**) 50MZ_2_ after last stage of thermal treatment at 1400 °C. The insets represent corresponding higher magnification images, highlighting particles shape and size.

Figure 7 presents the SEM micrographs of the 30MZ_2_ (Fig. 7a) and 50MZ_2_ (Fig. 7b) after high-temperature thermal treatment at 1400 °C. A granular shape of the hybrid nanocomposite can be observed in both samples, with a good distribution of the particles, as seen better in the lower magnification images. Nevertheless, in 30MZ_2_ sample (Fig. 7a) the average particles size is noticeably smaller than the one observed in 50MZ_2_ sample (Fig. 7b), due to the different rates of soldering process between particles during the thermal treatment for each sample, as previously discussed and as also suggested by smaller crystallite size ($D_{ef}$) for the 30MZ_2_ sample (see also Table 2).

In addition, the particles with rectangular facets noticeable in both samples (especially in the higher magnification insets), may suggest the hybrid nanocomposite took over the cubic crystal structure of TiN, as pointed out by the XRD spectra, too (see Fig. 6). Not in the least, the predominantly bigger rectangle shape of grains in 50MZ_2_ sample and mostly with smaller rounded particles in 30MZ_2_ sample sustained as well the values for the structural parameters given in Table 2 and the corresponding discussion.

In order to confirm the results from morpho-structural characterization, the elemental composition of samples 30MZ_2_ and 50MZ_2_ was measured by EDAX technique. The analysis of the recorded data shows for the sample 30MZ_2_ an atomic concentration of Ti 41.49 at.%, N 50.11 at.%, C 7.68 at.%, and O 0.72 at.%, respectively, and in case of the sample 50MZ_2_ the elemental composition is Ti 55.14 at.%, N 39.48 at.%, C 4.55 at.%, and O 0.83 at.%. Thus, the EDAX measurements further support the TiN formation from hybrid nanocomposite by CRN reaction, with impurities of carbon and oxygen in different ratio for each sample.

## Conclusion

Hybrid nanocomposite based on mesoporous TiO_2_/acrylonitrile monomers with different content of inorganic phase, were explored for the synthesis of TiN by CRN reaction. The successful syntheses of PAN and hybrid nanocoposite were proved FT-IR spectroscopy results. TGA results established the conditions and stages of thermal treatments used for TiN synthesis. The structural analysis based on XRD analysis of the samples thermal treated at 1400 °C in nitrogen atmosphere highlighted the formation of crystalline TiN phase as main phase along with some traces of impurities in the form of amorphous graphite and amorphous graphite oxide. Our results show that the dispersion of the inorganic phase in the hybrid nanocomposite affects the purity of the final product. Thus, further work will be focused on the investigation of different synthesis approaches for mesoporous TiO_2_, which can improve the dispersion along with the optimization of the parameters of the CRN reaction. Morphological characterization was performed by SEM and the results presented a granular distribution in hybrid nanocomposites with different size and shape of particles for each sample. SEM micrographs along with elemental composition from EDAX confirmed the observations made on structural properties obtained from XRD analysis. According to these results, the proposed method for TiN synthesis, relying on the formation of hybrid nanocomposites from mesoporous TiO_2_/acrylonitrile, represents an alternative amiable ammonia-free pathway of producing TiN-based materials with specific properties that can be tailored to a wide range of applications.

## Methods

### Materials

All chemicals were purchased from Sigma-Aldrich and they were used as purchased, without further purification unless specifically mentioned. Mesoporous anatase TiO_2_ (indexed as MZ_2_), used in this study, was received from National Institute for Research and Development in Electrochemistry and Condensed Matter (INCEMC). The inorganic matrix was obtained by a sol–gel process and Pluronic P−127 as a surfactant. The received powder has Brunauer–Emmett–Teller (BET) surface area of ~79 m^2^/g, pore surface area of   90 m^2^/g , pore diameter in desorption of ~9 nm and pore volume (measured ATP/P_0_ = 0.99) of ~0.2 cm^3^ /g , respectively. As a mesoporous material, the average pore diameter of titanium dioxide showed values above the 2 nm limit. The hybrid composites, 30MZ_2_ and 50MZ_2_, (where xMZ_2_- represents the weight percentage of TiO_2_ from inorganic–organic hybrid nanostructures) were obtained by radical polymerization of a vinyl monomer (AN) in pre-synthesized porous TiO_2_. Two different content of inorganic phase were used for hybrid nanocomposite synthesis, 30% and 50% respectively. The polymer composites were prepared by an original approach, consisting of two steps: (1) impregnation/adsorption of the AN in the TiO_2_ pores assisted by ultrasonication and (2) polymerization-assisted ultrasonication of the AN. Similar procedures were applied for the polymerization of AN alone, used as reference.

### Conversion of hybrid nanocomposites to TiN by CRN reaction

In order to obtain TiN ceramic materials, the hybrid nanocomposites have been subsequently subjected to four consecutive stages of a thermal treatment under nitrogen gas flow at 290 °C, 550 °C, 1000 °C and 1400 °C, respectively. The first three stages of the thermal treatment were related to the carbonization of PAN layer deposited in pre-synthesized mesoporous TiO_2_, whilst the last one involved the CRN process. It is well-known that the stabilization process is an important stage of the thermal treatment for producing carbon materials from PAN precursors. As reported elsewhere, the stabilization process of PAN took place at temperatures between 180 °C and 300 °C^35^^,^^26^. The process is characterized by different chemical and physical changes due to the cyclization, dehydrogenation, and oxidation reactions, where PAN precursors are converted to cyclized ladder structures that are the basic intermediates for the formation of the turbostratic graphite-like structure^35^^,^^21^. If the stabilization process took place in nitrogen atmosphere, intramolecular cyclization is the dominant reaction^35^^,^^36^. Thermal treatment at temperatures lower than 700 °C corresponds to the pre-carbonization process, where the chain scissions reaction dominates and the carbonization of PAN layers take place at temperatures higher than 800 °C^26^. Based on both, the above considerations and the results obtained from Thermal Gravimetric Analysis (TGA), our thermal treatment for PAN layer carbonization includes three stages: 290 °C, 550 °C and 1000 °C. Thus, the hybrid nanocomposites were thermal treated at 290 °C with 5 °C/min heating rate and 2h holding time, followed by subsequent thermal treatment at 550 °C with 5 °C/min heating rate and 4h holding time, under nitrogen gas flow. In the following stage, the samples were thermal treated at 1000 °C with 5 °C/min heating rate with 2h holding time under nitrogen flow. As reported in the references^12^^,^^15^, a more intimate contact of the reactants significantly accelerates the reaction, especially in its initial stages. The parameters used in our work for CRN reactions took in account the reported results that show the kinetics of TiO_2_ reduction represented as the rate of CO evolution in time during carbothermal reduction and nitriding of TiO_2_ reached the maximum value for samples treated at 1395 °C (with 10 °C/min as heating rate), followed by a rapid decrease to the values close to zero, after 2h, which was assumed to correspond to the formation of thermodynamically stable compounds^15^. Thus, in the present work we analyzed the possibility to use a better interface between the organic and inorganic phases given by the hybrid nanocomposites based on mesoporous TiO_2_/acrylonitrile as precursors for the CRN reaction, using the isothermal temperature of 1400 °C, with a heating rate of 10 °C/min and isothermal heating time of 2h. In all stages, the furnace was then cooled freely at the room temperature under controlled atmosphere.

### Characterization

Fourier Transform Infrared (FTIR) spectroscopy analysis of the samples was recorded on a Nicolet Summit FTIR Spectrometer equipment using 16 scans in the range of 550–4000 cm^−1^, with a resolution of 4 cm^−1^. The samples were analyzed using the ATR accessory, on a ZnSe crystal. The thermogravimetric analysis (TGA) of the polymer’s inorganic matrix, polymer and hybrid nanocomposites were recorded on TA Instruments Q500 equipment, as follows: a sample quantity (around 3 mg) was heated in the temperature range of 20–700 °C, with a speed heating of 10 °C/min. The analysis was performed in a nitrogen atmosphere, the gas flow in the furnace being 10 ml/min. Differential thermal analysis (DTA) provides detailed information on the polymer decomposition temperature. The morphology of the samples after CRN process was investigated by Scanning Electron Microscopy (SEM), using a Tescan Vega XMU-II microscope operating at 30 kV with a detection chain for secondary electrons. Energy Dispersive X-ray Analysis (EDAX) data were acquired with the Apreo S LoVac Scanning Electron Microscope using an accelerating voltage of 10kV. Ultimately, the starting materials and synthesized samples were characterized by X-ray diffraction (XRD) using a Bruker D8 Discover diffractometer ($\hbox {CuK}_\alpha$= 1.54 (Å) in Bragg–Brentano theta–theta geometry. The scattered X-ray photons from samples were recorded in the 2$\theta$ range of 10°–100° with a scanning rate of 0.04°/s at room temperature (unless otherwise mentioned). Rietveld analysis was then performed to determine the structural parameters of TiN, using Materials Analysis Diffraction (MAUD) program version 2.94.

## Data Availability

All data generated or analyzed during this study are included in the article.
